# Genomic Investigation Reveals Highly Conserved, Mosaic, Recombination Events Associated with Capsular Switching among Invasive *Neisseria meningitidis* Serogroup W Sequence Type (ST)-11 Strains

**DOI:** 10.1093/gbe/evw122

**Published:** 2016-06-11

**Authors:** Mustapha M. Mustapha, Jane W. Marsh, Mary G. Krauland, Jorge O. Fernandez, Ana Paula S. de Lemos, Julie C. Dunning Hotopp, Xin Wang, Leonard W. Mayer, Jeffrey G. Lawrence, N. Luisa Hiller, Lee H. Harrison

**Affiliations:** ^1^Infectious Diseases Epidemiology Research Unit, University of Pittsburgh; ^2^Public Health Dynamics Laboratory, Graduate School of Public Health, University of Pittsburgh; ^3^Molecular Genetics Laboratory, Public Health Institute of Chile, Santiago, Chile; ^4^Department of Bacteriology, Instituto Adolfo Lutz (IAL), São Paulo, Brazil; ^5^The Institute for Genome Sciences, University of Maryland School of Medicine, University of Maryland, Baltimore; ^6^Meningitis and Vaccine Preventable Diseases Branch, Division of Bacterial Diseases, National Center for Immunization and Respiratory Diseases, Centers for Disease Control and Prevention, Atlanta, Georgia, USA; ^7^Department of Biological Sciences, University of Pittsburgh; ^8^Department of Biological Sciences, Carnegie Mellon University, Pittsburgh, Pennsylvania, USA

**Keywords:** capsule switch, horizontal gene transfer, serogroup W135

## Abstract

*Neisseria meningitidis* is an important cause of meningococcal disease globally. Sequence type (ST)-11 clonal complex (cc11) is a hypervirulent meningococcal lineage historically associated with serogroup C capsule and is believed to have acquired the W capsule through a C to W capsular switching event. We studied the sequence of capsule gene cluster (*cps*) and adjoining genomic regions of 524 invasive W cc11 strains isolated globally. We identified recombination breakpoints corresponding to two distinct recombination events within W cc11: A 8.4-kb recombinant region likely acquired from W cc22 including the sialic acid/glycosyl-transferase gene, *csw* resulted in a C→W change in capsular phenotype and a 13.7-kb recombinant segment likely acquired from Y cc23 lineage includes 4.5 kb of *cps* genes and 8.2 kb downstream of the *cps* cluster resulting in allelic changes in capsule translocation genes. A vast majority of W cc11 strains (497/524, 94.8%) retain both recombination events as evidenced by sharing identical or very closely related capsular allelic profiles. These data suggest that the W cc11 capsular switch involved two separate recombination events and that current global W cc11 meningococcal disease is caused by strains bearing this mosaic capsular switch.

## Introduction

Polysaccharide capsule is the most important virulence determinant of *Neisseria meningitidis*, a normal commensal of the human nasopharynx that occasionally causes invasive meningococcal disease (IMD) (Stephens 2009). Even though un-encapsulated meningococci are common in asymptomatic carriage, they are very rarely associated with IMD (Mackinnon et al. 1993). The capsule is the target of most meningococcal vaccines and its biochemical and genetic properties form the basis of classifying meningococci into capsular groups (serogroups). Out of 12 capsular groups, groups A, B, C, W, X and Y cause almost all cases of IMD (Stephens 2009).

Sequence type (ST)-11 clonal complex (cc11), a hypervirulent meningococcal lineage, is a leading cause of IMD across all six continents (Caugant 1998; Harrison et al. 2009). Cc11 is a highly genetically diverse lineage associated with IMD caused by capsular serogroups C, B, W, and less commonly serogroup Y (Caugant 1998; Lucidarme et al. 2015). Serogroup C cc11 strains were associated with endemic IMD cases beginning in the 1960s, caused epidemics in the African “meningitis belt” in the 1970s, and small outbreaks globally (Broome et al. 1983; Caugant 1998; Halperin et al. 2012). Serogroup W cc11 was uncommon among IMD strains in the 1970s–1990s (Mustapha et al. 2016). The first epidemic of W cc11 occurred in Mecca, Saudi Arabia among Hajj pilgrims and their close contacts in 2000 (Taha et al. 2000). Since 2000, W cc11 has emerged as a leading cause of epidemic IMD in the meningitis belt, whereas endemic clusters have emerged in South America, Middle East, Europe, and China in 2000–2015 (Mustapha et al. 2016).

The capsule gene cluster, *cps*, is a 24-kb genetic island horizontally acquired by *N meningitidis* that is not present in closely related *N. lactamica* and *N. gonorrhoeae* (Dunning Hotopp et al. 2006; Lam et al. 2011). A recent study described *cps* architecture and gene content (Harrison et al. 2013). All known meningococcal serogroups had *cps* containing five gene regions involved in capsule synthesis, transport and assembly (*cps* regions A–E) (Harrison et al. 2013). Serogroups B, C, W and Y have capsules containing sialic acid residues (Swartley et al. 1997; Harrison et al. 2013; Romanow et al. 2014). The transferase gene, located in region A, dictates linkages and thus determines serogroup phenotype (Claus et al. 1997; Harrison et al. 2013). Capsular serogroup diversity among meningococci within the same clonal complex is driven by “capsular switching”—lateral exchange of capsule biosynthetic genes through homologous recombination (Swartley et al. 1997; Kong et al. 2013). For a recombination event to result in change of capsular phenotype, allelic changes must include region A genes, particularly the transferase gene (Swartley et al. 1997; Romanow et al. 2013). Meningococcal capsular switching is relatively common and has been associated with the emergence and persistence of IMD (Swartley et al. 1997; Beddek et al. 2009; Harrison et al. 2010; Castineiras et al. 2012). Capsular switching is also of public health relevance given that majority of meningococcal vaccines target the capsule (Cohn and Harrison 2013).

Recent phylogenomic study of 750 meningococcal cc11 isolates (Lucidarme et al. 2015) revealed a highly complex population structure with extensive genetic diversity among cc11 strains. Serogroup C cc11 strains formed several clusters linked to multiple epidemiological instances of serogroup C disease; B strains were interspersed among serogroup C clusters; whereas serogroup W formed a distinct phylogenetic branch that was not interspersed with either B or C strains. Additionally, W cc11 lineage was genetically heterogeneous, containing diverse phylogenetic clusters associated with the following: (1) the Hajj clone, (2) endemic W cc11 strains unrelated the Hajj clone in Africa, and (3) a distinct South American W cc11 strain in Brazil, Argentina, Chile that has also emerged in United Kingdom (Lucidarme et al. 2015; Mustapha et al. 2015, 2016).

Given the preponderance of serogroup C among cc11 strains, it is regarded by some to be the founder capsule type with serogroups W, B and Y representing capsular switch variants (Kelly and Pollard 2003; Mueller et al. 2006; Mustapha et al. 2016). However, the direction and exact genes involved in capsular switching have not been established. The purpose of this study is to analyze capsular gene sequences from a global collection of strains to characterize the capsular switching events associated with W cc11.

## Results

### W cc11 Reference Strain, M7124 Contains Mosaic Recombinant Sequences

**Fig. 1.**
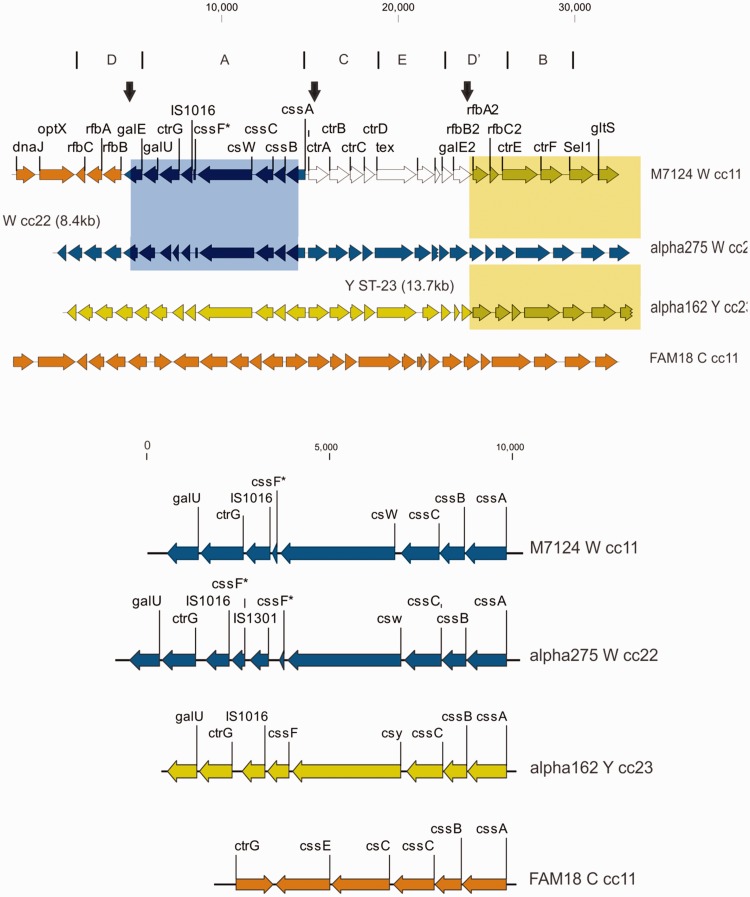
Top panel: Capsule gene cluster (*cps*) for W cc11 reference strain M7124 and corresponding genes for reference strains α-275 (W ST-22), α-162 (Y ST-23) and FAM18 (C ST-11). *Cps* is comprised of five regions A–E; synteny and gene content is divergent within region A (see below) but conserved within regions B–E among listed reference strains. Two recombination events are depicted: (1) an 8.4-kb W cc22 donor fragment (blue rectangle) corresponding to α-275 reference genome. This recombinant segment includes all region A genes; (2) a 13.7-kb Y cc23 donor fragment corresponding to α-162 reference genome (yellow rectangle). This recombinant fragment includes parts of *cps* region D′, entire region B and adjoining sequences downstream of *cps.* Downstream end of this recombinant segment, outside of cps, is truncated for clarity; Vertical (black) arrows indicate recombination breakpoints within *galE, cssA* and *rfbA2*. Bottom panel: Gene content within *cps* region A among meningococcal reference strains. *cssA-C* genes are syntenic in all strains; sialyl transferase gene is divergent with a 1.5-kb *csc* in serogroup C, whereas the *csw* and *csy* genes for serogroups W and Y are both 3.1 kb in size. In addition, W ST-11 further differs from C ST-11 by having truncated O-acetyl transferase, *cssF* gene (*) fragments, one or more IS elements, reverse orientation of *ctrG* and presence of the *galU* gene.

The *cps* locus of M7124, a previously characterized W ST11 Hajj clone reference strain (Mustapha et al. 2015), is 27 kb in length with genes organized into regions D–A–C–E–D′–B (fig. 1) similar to that described for other meningococcal lineages (Harrison et al. 2013).

**Fig. 2.**
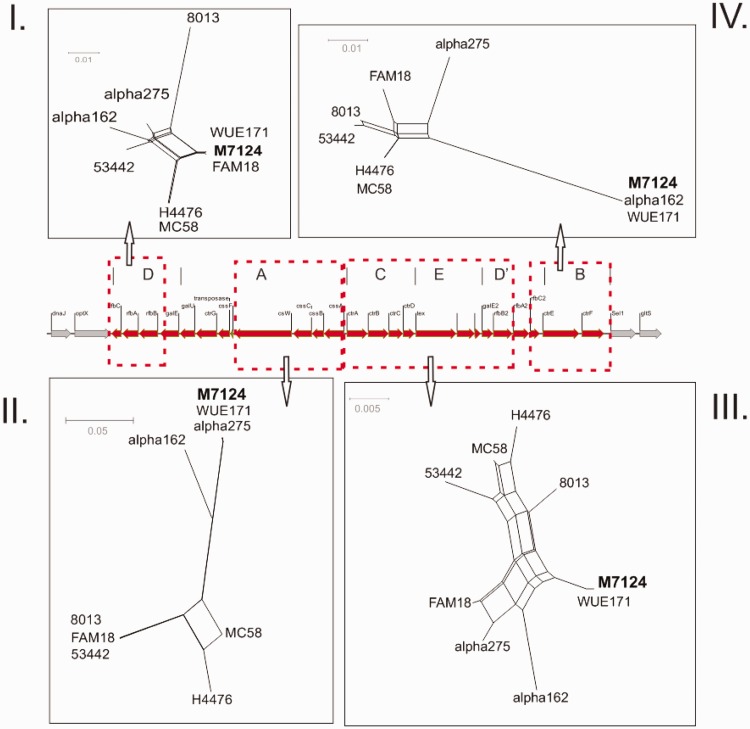
Phylogenetic relationships of M7124, W cc11 to *cps* from meningococcal reference genomes. Aligned *rfbC1-rfbB1* genes (region D, panel I) demonstrate very close phylogenetic relationship between W cc11 (M7124 and WUE171) and serogroup C cc11 (FAM18). Phylogeny of aligned region A genes (panel II) shows that W cc11 (M7124 and WUE171) cluster with serogroup W cc22 (α-275). Across regions C, E and upstream parts of region D′ (panel III) W cc11 sequences do not cluster with any of the reference strains. While aligned region B sequences (panel IV) demonstrate clustering between W cc11 and Y cc23 (α-162). WUE171 serogroup W cc11; FAM18, C cc11; MC58, B cc32; H4476, B cc41/44 α-275, W cc22; 8013 C cc18; 53442 C cc4821; and α-162 Y cc23. Dotted red boxes depict margins of sequence alignments used to generate corresponding phylogenetic network.

**Fig. 3.**
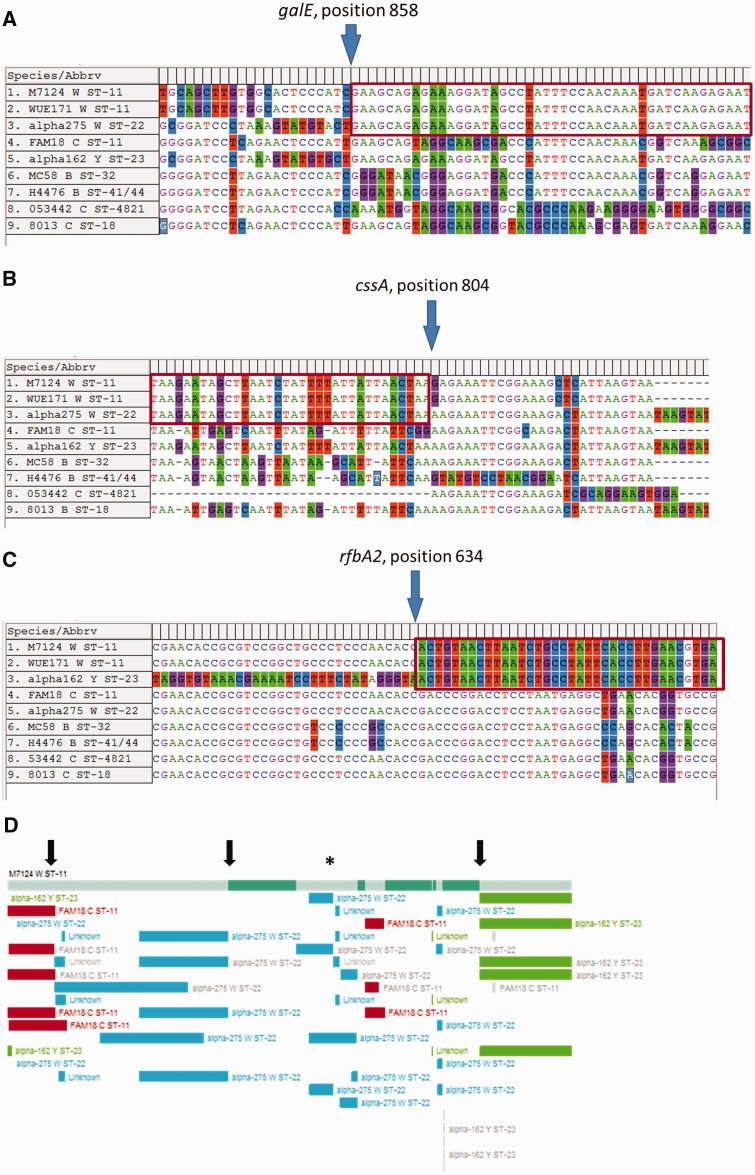
Recombination breakpoints within M7124 capsule. (*A*) Sequence alignment of *galE* genes showing recombination breakpoint at position 858 relative to W cc22 strain, α-275. (*B*) Sequence alignment of *cssA* genes demonstrating breakpoint at position 804 relative to α-275. (*C*) Sequence alignment of *rfbA2* and *rfbC2* showing recombination breakpoint within *rfbA2* position 634. (*D*) Recombination breakpoints detected by at least one of seven methods included in RDP v4.56. The topmost horizontal bar represents M7124 *cps* sequence; three black arrows correspond to locations of breakpoints identified in (*A–C*) above; asterisk marks location of multiple small (0.1–1.5 kb) putative mosaic recombinant segments that were not supported by visual examination of aligned sequences (see main text). Topmost horizontal bar represents W cc11; colored boxes represent donor sequences from Y cc23 (green), W cc22 (blue) and C cc22 (red).

When M7124 *cps* sequences were compared with other *cps* reference sequences, a number of abrupt changes in sequence similarity consistent with distinct recombination events were identified. First, M7124 shares very high sequence similarity with the serogroup W cc22 reference strain α-275 over a 8.4-kb segment that includes all *cps* region A genes (figs. 1 and 2, panel II). Only three nucleotide differences are evident between M7124 and α-275 over the entire 8.4-kb segment (fig. 1). Flanking this segment of high sequence similarity are two areas of sharp divergence in sequence similarity in keeping with recombination breakpoints at nucleotide positions 858 and 804 on *galE* and *cssA* genes, respectively (fig. 3*A* and *B*). In contrast, there were >1255 and 52 nucleotide differences between M7124 and α-275 within 5-kb upstream and downstream of the respective recombination breakpoints (data not shown). A recombination event with W cc22 as the likely donor strain is supported by alignment of *galE* and *cssA* gene sequences (fig. 3*A* and *B*). Phylogenetic networks (fig. 2, panel II) of meningococcal reference sequences across *cps* region A demonstrate that M7124 sequences were phylogenetically indistinguishable from corresponding W cc11 (WUE171) and W cc22 (α-275) strains, providing further evidence of this recombination event. Genes involved in this recombination event (listed from 5′ to 3′) included *galE* (partial), *galU, ctrG, cssF, csw, cscC, cssB and cssA* (partial) (fig. 1). The transferase gene, *csw* acquired within this recombination is responsible for capsular serogroup W phenotype (fig. 1).

To further investigate whether W cc22 was the potential donor lineage, we assessed the prevalence of the recombinant sequence among 55 W ST-22 genome sequences in the PubMLST database (http://pubmlst.org/neisseria/). Eighty percent (44/55) of W ST-22 strains shared a common *cssB-C*, *csw* and *ctrG* allelic profile differing from M7124 by only two nucleotide substitutions within *csw*. These data demonstrate that this upstream recombination is predominant among W cc22 strains.

Second, M7124 shares a 13.7-kb region of very high sequence similarity with the serogroup Y cc23 strain, including 4.5 kb of *cps* and 9.2 kb downstream of the *cps* cluster (fig. 1). There was 100% nucleotide sequence identity between M7124 and Y cc23 reference strain α-162 over this region. This region of 100% sequence identity contrasts with the significant sequence divergence observed within the sequences flanking the 13.7-kb region. The upstream breakpoint corresponds to position 634 of *rfbA2* gene (figs. 1, 2, and 3*C*), whereas a downstream breakpoint is located outside the *cps* cluster at the 5′ end of *pykA* gene (data not shown). The entire region B (*ctrE* and *ctrF*) and parts of region D′ (*rfbA2* and *rfbC2*) were part of this recombinant segment (figs. 1 and 2) which also extends beyond *cps* and includes the genes encoding a sodium/glucose transport protein (*gltS*), a pyruvate kinase (*pykA*), tetratricopeptide repeat (*sel1*), and several hypothetical proteins. Phylogenetic network analysis of aligned region B among reference *cps* sequences demonstrates that M7124, WUE171 (W cc11) and α-162 (Y cc23) formed a phylogenetic cluster that was distinct from other meningococcal reference strains (fig. 2, panel IV).

We also compared M7124 *ctrE* and *ctrF* genes to 201 Y cc23 genome sequences from the PubMLST database. Over 88% (177/201) of all Y cc23 isolates shared identical *ctrE* and *ctrF* gene sequences with M7124 demonstrating that this allelic combination is predominant among Y cc23 strains. Taken together these data support the presence of a 13.7-kb recombinant segment that extends downstream of *cps* with Y cc23 as the most likely donor lineage.

All three recombination break points within *cps* were confirmed by automated detection of recombination using RDP v4.56 (fig. 3*D*) (Martin et al. 2010). In addition, one-third 3.0-kb recombinant fragment within *cps* region C involving *ctrB–D* genes was suggested by RDP analysis. This putative recombinant fragment was ruled out following visual inspection of aligned sequences that found 39 nucleotide differences between M7124 and the putative donor, α-275 (W ST-22).

### M7124 Recombination Pattern Is Highly Conserved among Global W cc11 Strains Isolated from 1970 to 2014

**Fig. 4.**
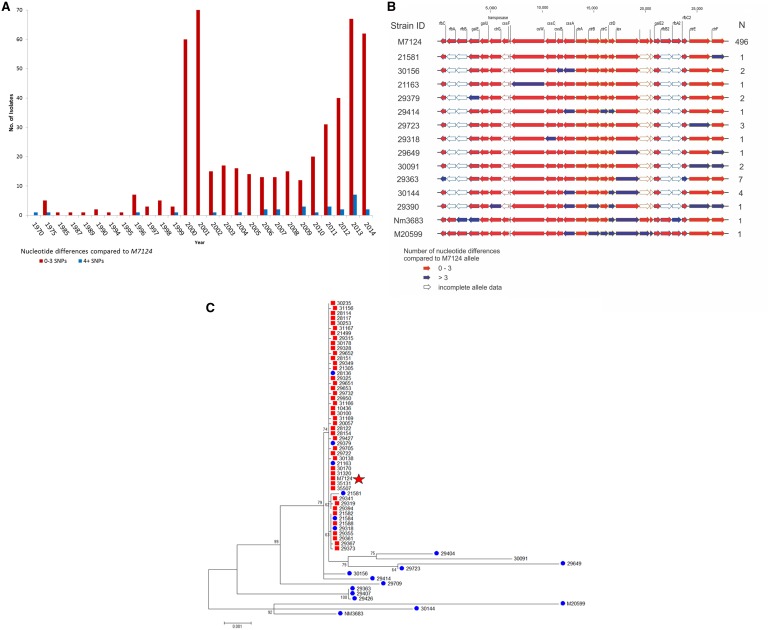
Summary of W cc11 *cps* allelic profiles. (*A*) Graphic depiction of 524 *cps* allelic profiles relative to M7124. Red arrows: genes with ≤3 nucleotide differences; blue arrows: genes with >3 nucleotide differences; white boxes: genes with incomplete sequence data. Strain ID: representative strain within each *cps* allelic profile. N: number of isolates. (*B*) Distribution of W cc11 *cps* allelic profile relative to M7124 over time; Red bars: strains with ≤3 nucleotide differences; blue bars: strains with >3 nucleotide differences. (*C*) Maximum likelihood tree depicting phylogenetic relationship between selected W cc11 *cps* sequences. Red squares: strains with ≤3 nucleotide differences relative to M7124 (red star); blue dots: strains with ≥4 nucleotide differences.

To assess what proportion of W cc11 strains contained this M7124 mosaic recombination, sequence comparison of 16 *cps* alleles (*ctrA–G, cssA–C, csw, tex, galE, galE2, rfbC* and *rfbC2*) from 524 W cc11 strains isolated from 21 different countries was performed (supplementary table, Supplementary Material online). The majority of W cc11 strains (63.9%; 334/523) shared identical *cps* allelic profiles with M7124, whereas 31.0% (162/523) differed by ≤3 point mutations across all 16 *cps* genes. Thus, the majority (95%, 496/523) of sequenced W cc11 strains representing a global collection isolated from 1970 to 2014 shared the same *cps* gene structure as M7124 (fig. 4*A* and *B*). A phylogeny of contiguous *cps* gene sequences shows a single cluster highly related to M7124 (fig. 4*C*, red boxes). While some strains with >3 SNPs relative to M7124 belonged to the same cluster, the majority were distinct from M7124 (fig. 4*C*, blue dots).

**Fig. 5.**
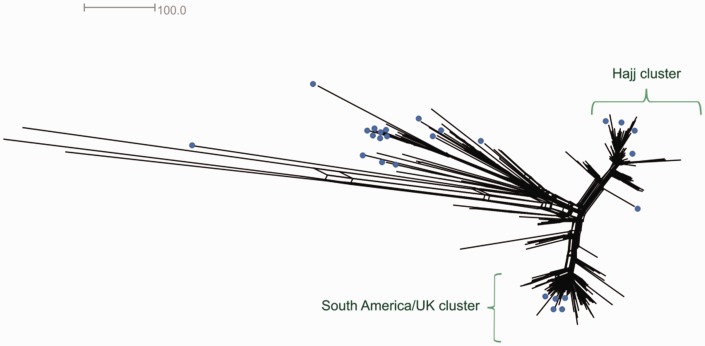
Core genome phylogenetic network of 524 W cc11 strains reconstructed using neighbor-net algorithm on SplitsTree v4. Twenty-eight strains with *cps* allelic shift relative to M7124 are represented by blue dots.

Twenty-eight isolates (28/523, 5.4%) with nonM7124 *cps* gene structure were further examined. All 28 isolates had one or more *cps* genes that exhibited *cps* allelic shift, defined as >3 nucleotide differences within a single gene (fig. 4a, blue boxes). Thirteen isolates (46.4%, 13/28) had allelic shift in a single *cps* gene, whereas the remaining 16 isolates had two to seven genes with allelic shift (fig. 4a,blue rectangles). Also, seven isolates collected from South Africa in 2006–2013 each had three genes with allelic shift and shared a common *cps* allelic combination suggesting geographically limited clonal spread (fig. 4*A* and supplementary table, Supplementary Material online). Twenty-two remaining strains with one or more allelic shifts were geo-temporally and phylogenetically diverse without any predominant allelic profile (fig. 4*B* and *C* and supplementary table S1, Supplementary Material online). Interestingly, all 28 isolates with *cps* allelic shift contained at least one of the two recombinant sequences identified in M7124. Core genome phylogenetic network of 524 W cc11 isolates demonstrated that strains with *cps* allelic shift were interspersed among strains with M7124 *cps* type (fig. 5, blue dots) in keeping with sporadic rather than clonal pattern of allelic shift.

There was no statistically significant difference in proportion of M7124 allelic type among W cc11 strains isolated in 1970–1999 compared with 2000–2014 (87.9% vs. 95.1%, *P* = 0.09). Likewise, excluding United Kingdom and South Africa strains does not change the proportion of isolates with M7124 *cps* allelic type (96.2% vs. 94.7% *P* = 0.6). These data suggest that observed dominance of M7124 *cps* allelic type among W cc11 isolates is unlikely to be a result of clonal expansion of the Hajj clone in 2000 or an artifact caused by the large proportion of isolates from United Kingdom and South Africa.

## Discussion

In this study, we present detailed characterization of the capsule gene locus, the primary meningococcal virulence determinant within the context of globally emergent hypervirulent serogroup W cc11 lineage. Our results suggest that W cc11 acquired the capsule polymerase (*csw*) gene along with other region A genes from a W cc22 strain and capsule translocation genes from a strain belonging to the Y cc23 lineage. Also, an overwhelming majority of W cc11 strains had *cps* allelic profiles very closely related to M7124 suggesting a shared recombinant capsule structure from a common ancestor. Strains with one or more divergent alleles were a very small minority but were observed sporadically from 1970 to 2014. These data are consistent with recent genome sequencing studies showing that all W cc11 strains belonged to a single lineage that is phylogenetically distinct from serogroup C and B cc11 lineages (Lucidarme et al. 2015). Also, these data raise the possibility of a multi-step C to W switch involving a serogroup Y strain. Detailed characterization of global C, B and Y capsular genes is needed to address these questions.

We propose that the Hajj clone and a vast majority of W cc11 strains acquired an 8.4-kb recombinant fragment from serogroup W cc22 lineage. This recombination event includes transferase gene (*csw*) that mediates formation of (α2→6) sialic acid and glucose heteropolymers characteristic of serogroup W capsule (Harrison et al. 2013). The observed 8.4 kb recombinant fragment in this study is consistent with the finding of a 9-kb allelic exchange of *cps* regions resulting in a serogroup B to C ST-32 capsular switch among isolates from a serogroup B epidemic in Oregon (Swartley et al. 1997); and a 12-kb recombination involving the entire *cps* region A and flanking genes within region D and C leading to a switch from serogroup A ST-7 to C in China (Wang et al. 2010). These studies highlight that even though allelic exchange within a single gene (sialyl-transferase) may be sufficient to cause capsular switching, large recombination events affecting several genes are common.

We also identified an additional recombination event involving genes associated with capsule translocation (*ctrE, ctrF*) from Y cc23 donor lineage. This recombination event did not involve capsule synthesis genes in region A and therefore had no corresponding change in capsular serogroup. However, even without an obvious change in capsular phenotype, such allelic exchanges outside region A could affect meningococcal virulence by altering capsule transport and modification (Tzeng et al. 2005). For example, *ctrE* is up-regulated during meningococcal invasion of human cells (Spinosa et al. 2007) so recombination within this gene has the potential to enhance virulence through enhanced intracellular survival.

The very high degree of sequence conservation among W cc11 capsule genes markedly contrasts with the substantial global and temporal variability in W cc11 disease incidence patterns (Mustapha et al. 2016), a lineage associated with both sporadic disease (Molling et al. 2001; Zhou et al. 2013) and epidemics (Taha et al. 2000; MacNeil et al. 2014). Meningococci are associated with extensive genetic diversity through homologous recombination, phase variation and point mutations (Holmes et al. 1999; Hao et al. 2011; Kong et al. 2013). In fact, our previous study demonstrated that the epidemic W cc11 strain, “Hajj clone,” and its descendants acquired virulence gene alleles outside of the *cps* cluster, through a set of unique recombination events affecting factor H binding protein (*fHbp*), nitric oxide reductase (*nor*) and nitrite reductase (*aniA*) genes (Mustapha et al. 2015). Taken together, these studies suggest that although a unique, mosaic, recombinant capsule locus is a common feature across most hypervirulent W cc11 strains, recombination across other genomic loci mediate changes in strain virulence over time.

To our knowledge, these data represent the first description of allelic coupling across a large number of *cps* genes. Such extensive allelic coupling was unexpected given that the horizontal transfer of a single *cps* region A gene allele (*csw*) from W cc22 was sufficient to cause phenotypic change from an ancestral C to W serogroup (Claus et al. 1997; Harrison et al. 2013; Swartley et al. 1997). Significance of the second recombination from Y cc23 could be to offset any fitness cost associated with the first (W cc22) recombination event. This is supported by the observation that W cc11 represents a rare example where a capsular switch strain has persisted for decades and spread to several continents, as compared with a majority of capsular switch strains that do not persist in the long term (Harrison et al. 2010; Barroso et al. 2013; He et al. 2014; Zhu et al. 2015). Dominance of single *cps* allelic type is consistent with the “genocloud” concept in which a set of mutually co-adapted sets of genes persist despite presence of a few, mostly transient, escape variants (Gupta et al. 1996; Zhu et al. 2001). In general, DNA uptake sequences (Duffin and Seifert 2010) and restriction modification systems (Budroni et al. 2011; Tibayrenc and Ayala 2015) are known to affect the rate, extent and donor specificity of recombination in *Neisseria*. However, the precise role of these factors in capsular switching is yet to be established.

A limitation of our study is that we were unable to identify intermediate *N. meningitidis* isolates that had only one of the two recombinant sequences. These intermediate strain(s) may have lacked the biologic fitness to survive for several decades (Zhu et al. 2001). Also, our method of assigning donor lineages is, by design, conservative in that it only assigns events when there is a match that spans at least 2 kb and if the identified recombinant allele is present in a majority of putative donor lineage isolates in the *Neisseria* genome database. This approach could miss smaller recombination fragments that may have given rise to novel, mosaic, genes matching neither the donor nor the ancestral allele. In addition, a vast majority of *Neisseria* genome sequences, including most of the 524 W cc11 strains in this study, were isolated from invasive disease cases. Therefore, some of the genetic diversity among carriage strains could possibly have been missed.

In summary, we have demonstrated that the W cc11 lineage likely arose through recombination from ancestral serogroup W cc22 and serogroup Y cc23 donor strains. Remarkably, strains with this mosaic recombination have persisted as the dominant global W cc11 strain from 1970 to 2014 despite emergence of a phylogenetically distinct epidemic Hajj clone (Mustapha et al. 2016) and genetically diverse endemic case clusters globally.

## Material and Methods

### Study Isolates

A total of 528 serogroup W cc11 genome sequences with corresponding capsule gene allele designations were identified from PubMLST (www.pubmlst.org/neisseria, last accessed September, 2015), a database that captures *Neisseria* genetic diversity by assigning allele numbers for all *Neisseria* genes. Four isolates (*19369, 19377, 19379*, and *19381*) were excluded because they had missing data for a majority of *cps* alleles, whereas *cps* allele designations for 524 isolates were included in the study (supplementary table, Supplementary Material online). All but 1 of the 524 W ST-11 strains were isolated from patients with IMD. M7124, identified in Saudi Arabia during the Hajj 2000 epidemic, is a well characterized reference strain for the W cc11 lineage (GenBank accession number, CP009419) sequenced using single molecule, real-time technology (SMRT, www.pacb.com) into a single contiguous sequence (contig) and is used in this study as W cc11 *cps* reference genome (Mustapha et al. 2015).

### Identification of Recombination Donors and Breakpoints for M7124

To determine the organization of *cps* genes *cps* sequences were extracted for the M7124 and compared with eight previously described *cps* sequences for reference meningococcal strains (Harrison et al. 2013) using SplitsTree v4 (Huson and Bryant) phylogenetic networks and visual inspection of sequence alignments. These strains were selected because they represent the best characterized *cps* gene sequences with no assembly gaps or missing data. Also, potential recombination donor lineages were assessed by querying *Neisseria* genome sequences in PubMLST and GenBank databases to determine closest matches outside of the W cc11 lineage.

Our method of assessing recombination and assigning donor lineage is, by design, conservative based on the following criteria. A strain was considered a potential donor for *M7124* capsular genes if: (1) there is a match that spans at least 2 kb, (2) less than one SNP difference per 1000 base pairs between donor and recombinant gene sequences, and (3) the identified recombinant gene allele is present in a majority of putative donor lineage isolates on the *Neisseria* genome databases. Recombination breakpoints were identified from sequence alignments as points of abrupt change in sequence similarity between *M7124* and a potential donor strain (Swartley et al. 1997). Recombination break points identified by visual inspection of *cps* sequence alignments were re-assessed using Recombination Detection Program (RDP) v4.56 (Martin et al. 2010). Putative recombination breakpoints identified by ≥1 of 7 algorithms (RDP, Chimaera, Bootscan, 3Seq, GENECONV, MaxChi and SiScan) within *cps* sequence alignment of four meningococcal strains, specifically M7124, C ST-11 (FAM18), Y ST-23 (α-162), and W ST-22 (α-275), were compared with visually detected break points.

### Comparison of *cps* Alleles within W cc11 Lineage

M7124 *cps* gene sequences were compared with 524 W cc11 strains with genome sequence data isolated from 21 different countries. Majority (93.5%, 489/523) were isolated in 2000–2014 with the remaining 34 isolated in 1970–1999. Most isolates were from United Kingdom (330/523, 63.1%), South Africa (116/523, 22.2%) or the African “meningitis belt” (34/523, 6.5%; supplementary table, Supplementary Material online) (Lapeyssonnie 1968). Allelic variants for 16 *cps* genes (*ctrA-G, cssA-C, csw, tex, galE, galE2, rfbC* and *rfbC2*) that had complete sequences for ≥80% of the study isolates were downloaded. Allele comparisons of *rfbA* and *rfbB* genes was not performed due to missing sequence data among 91% (479/524) and 79% (416/524) of total isolates, respectively. Also, gene elements not curated by PubMLST including insertion sequences, putative fragments in close proximity to insertion sequences (specifically, *cssF* gene fragments) and two pseudo-genes (putative methyl transferases) within region E were excluded from further analyses. Over 70% of isolates (368/524) had complete sequence data for all 16 *cps* genes, 22.3% (117/524) had missing data for one or two genes, whereas 39 (7.3%) had missing data for three to five genes. *galE2* (99/524, 19.9%), and *galE* (94/524, 17.9%) genes had the highest frequency of missing data (supplementary table, Supplementary Material online).

Isolates that had either an identical allelic profile to M7124 or had one to three nucleotide differences across all nonmissing *cps* genes were considered the same *cps* gene structure as M7124. Isolates with >3 nucleotide differences across all *cps* genes were further examined to identify whether nucleotide differences clustered into one or more genes. Any gene that differed from M7124 by >3 nucleotide was defined as an “allelic shift” suggestive of a unique recombination in that particular gene in a given isolate compared with M7124. Presence and total number of allelic shifts were assessed for each isolate.

Phylogenetic tree of all 524 W cc11 isolates was obtained using the core genome MLST (cgMLST) protocol from the *Neisseria* genome PubMLST database (http://pubmlst.org/neisseria/) and visualized on SplitsTree v4 (fig. 5). This method generates a neighbornet phylogenetic network based on allelic profiles across 1605 universally present meningococcal genes (Huson and Bryant 2006; Bratcher et al. 2014). *Cps* sequences were then concatenated and aligned for a subset of 65 isolates selected to capture capsular allelic diversity within W cc11 (fig. 3). All 29 isolates with ≥4 nucleotide differences across *cps* gene alleles and 36 isolates, representative of the geo-temporal diversity of isolates within zero to three nucleotide differences from M7124, were selected for this phylogenetic analysis. At least one isolate was selected from every *cps* allelic combination and strains that were isolated >5 years apart or from different countries were included even if they shared allelic profile with another included strain. MEGA v5.2 (Tamura et al. 2011) and CLC genomics workbench v8.0 (www.clcbio.com) were used to generate sequence alignments and maximum likelihood phylogenetic trees under the under the Hasegawa, Kishino and Yano model of evolution, Γ-distribution of substitution rates and invariant sites (HKY + Γ+I) with 500 bootstrap replicates.

## Supplementary Material

Supplementary table S1 is available at *Genome Biology and Evolution* online (http://www.gbe.oxfordjournals.org/).

Supplementary Data

Supplementary Data

## References

[evw122-B1] Barroso DE, 2013 *Neisseria meningitidis* ST-11 clonal complex bearing capsule serogroups B, C, or W in Brazil. J Infect. 66:547–550. 23321151 10.1016/j.jinf.2013.01.002 PMC4646077

[evw122-B2] Beddek AJ Li MS Kroll JS Jordan TW Martin DR. 2009 Evidence for capsule switching between carried and disease-causing *Neisseria meningitidis* strains. Infect Immun. 77:2989–2994. 19451248 10.1128/IAI.00181-09 PMC2708544

[evw122-B3] Bratcher HB Corton C Jolley KA Parkhill J Maiden MC. 2014 A gene-by-gene population genomics platform: de novo assembly, annotation and genealogical analysis of 108 representative *Neisseria meningitidis* genomes. BMC Genomics 15:1138. 25523208 10.1186/1471-2164-15-1138 PMC4377854

[evw122-B4] Broome CV, 1983 Epidemic group C meningococcal meningitis in Upper Volta, 1979. Bull World Health Organ. 61:325–330. 6345014 PMC2536118

[evw122-B5] Budroni S, 2011 *Neisseria meningitidis* is structured in clades associated with restriction modification systems that modulate homologous recombination. Proc Natl Acad Sci U S A. 108:4494–4499. 21368196 10.1073/pnas.1019751108 PMC3060241

[evw122-B6] Castineiras TM, 2012 Capsular switching in invasive *Neisseria meningitidis*, Brazil. Emerg Infect Dis. 18:1336–1338. 22840713 10.3201/eid1808.111344 PMC3414017

[evw122-B7] Caugant DA. 1998 Population genetics and molecular epidemiology of *Neisseria meningitidis.* Apmis 106:505–525. 9674888

[evw122-B8] Claus H Vogel U Muhlenhoff M Gerardy-Schahn R Frosch M. 1997 Molecular divergence of the *sia* locus in different serogroups of *Neisseria meningitidis* expressing polysialic acid capsules. Mol Gen Genet. 257:28–34. 9439566 10.1007/pl00008618

[evw122-B9] Cohn AC Harrison LH. 2013 Meningococcal vaccines: current issues and future strategies. Drugs 73:1147–1155. 23839656 10.1007/s40265-013-0079-2

[evw122-B10] Duffin PM Seifert HS. 2010 DNA uptake sequence-mediated enhancement of transformation in *Neisseria gonorrhoeae* is strain dependent. J Bacteriol. 192:4436–4444. 20601472 10.1128/JB.00442-10 PMC2937394

[evw122-B11] Dunning Hotopp JC, 2006 Comparative genomics of *Neisseria meningitidis*: core genome, islands of horizontal transfer and pathogen-specific genes. Microbiology 152:3733–3749. 17159225 10.1099/mic.0.29261-0

[evw122-B12] Gupta S, 1996 The maintenance of strain structure in populations of recombining infectious agents. Nat Med. 2:437–442. 8597954 10.1038/nm0496-437

[evw122-B13] Halperin SA, 2012 The changing and dynamic epidemiology of meningococcal disease. Vaccine 30(Suppl 2):B26–B36. 22178525 10.1016/j.vaccine.2011.12.032

[evw122-B14] Hao W, 2011 Extensive genomic variation within clonal complexes of *Neisseria meningitidis.* Genome Biol Evol. 3:1406–1418. 22084315 10.1093/gbe/evr119 PMC3242501

[evw122-B15] Harrison LH, 2010 Population structure and capsular switching of invasive *Neisseria meningitidis* isolates in the pre-meningococcal conjugate vaccine era—United States, 2000-2005. J Infect Dis. 201:1208–1224. 20199241 10.1086/651505 PMC2838939

[evw122-B16] Harrison LH Trotter CL Ramsay ME. 2009 Global epidemiology of meningococcal disease. Vaccine 27(Suppl 2):B51–B63. 19477562 10.1016/j.vaccine.2009.04.063

[evw122-B17] Harrison OB, 2013 Description and nomenclature of *Neisseria meningitidis* capsule locus. Emerg Infect Dis. 19:566–573. 23628376 10.3201/eid1904.111799 PMC3647402

[evw122-B18] He B, 2014 CC4821 serogroup W meningococcal disease in China. Int J Infect Dis. 29:113–114. 25461240 10.1016/j.ijid.2014.08.022

[evw122-B19] Holmes EC Urwin R Maiden MC. 1999 The influence of recombination on the population structure and evolution of the human pathogen *Neisseria meningitidis.* Mol Biol Evol. 16:741–749. 10368953 10.1093/oxfordjournals.molbev.a026159

[evw122-B20] Huson DH Bryant D. 2006 Application of phylogenetic networks in evolutionary studies. Mol Biol Evol. 23:254–267. 16221896 10.1093/molbev/msj030

[evw122-B21] Jolley KA Maiden MC. 2010 BIGSdb: scalable analysis of bacterial genome variation at the population level. BMC Bioinformatics 11:595. 21143983 10.1186/1471-2105-11-595 PMC3004885

[evw122-B22] Kelly D Pollard AJ. 2003 W135 in Africa: origins, problems and perspectives. Travel Med Infect Dis. 1:19–28. 17291877 10.1016/S1477-8939(03)00019-X

[evw122-B23] Kong Y, 2013 Homologous recombination drives both sequence diversity and gene content variation in *Neisseria meningitidis.* Genome Biol Evol. 5:1611–1627. 23902748 10.1093/gbe/evt116 PMC3787668

[evw122-B24] Lam TT Claus H Frosch M Vogel U. 2011 Sequence analysis of serotype-specific synthesis regions II of *Haemophilus influenzae* serotypes c and d: evidence for common ancestry of capsule synthesis in Pasteurellaceae and *Neisseria meningitidis*. Res Microbiol. 162:483–487. 21513796 10.1016/j.resmic.2011.04.002

[evw122-B25] Lapeyssonnie L. 1968 [Comparative epidemiologic study of meningococcic cerebrospinal meningitis in temperate regions and in the meningitis belt in Africa. Attempt at synthesis]. Med Trop (Mars). 28:709–720. 5739513

[evw122-B26] Lucidarme J, 2015 Genomic resolution of an aggressive, widespread, diverse and expanding meningococcal serogroup B, C and W lineage. J Infect. 71(5):554–552. 10.1016/j.jinf.2015.07.007 PMC4635312 26226598

[evw122-B27] Mackinnon FG, 1993 Demonstration of lipooligosaccharide immunotype and capsule as virulence factors for *Neisseria meningitidis* using an infant mouse intranasal infection model. Microb Pathog. 15:359–366. 7912406 10.1006/mpat.1993.1085

[evw122-B28] MacNeil JR, 2014 *Neisseria meningitidis* serogroup W, Burkina Faso, 2012. Emerg Infect Dis. 20:394–399. 24571805 10.3201/eid2003.131407 PMC3944835

[evw122-B29] Martin DP, 2010 RDP3: a flexible and fast computer program for analyzing recombination. Bioinformatics 26:2462–2463. 20798170 10.1093/bioinformatics/btq467 PMC2944210

[evw122-B30] Molling P Backman A Olcen P Fredlund H. 2001 Comparison of serogroup W-135 meningococci isolated in Sweden during a 23-year period and those associated with a recent Hajj pilgrimage. J Clin Microbiol. 39:2695–2699. 11427599 10.1128/JCM.39.7.2695-2699.2001 PMC88215

[evw122-B31] Mueller JE Borrow R Gessner BD. 2006 Meningococcal serogroup W135 in the African meningitis belt: epidemiology, immunity and vaccines. Expert Rev Vaccines. 5:319–336. 16827617 10.1586/14760584.5.3.319

[evw122-B32] Mustapha MM, 2015 Genomic epidemiology of hypervirulent serogroup W, ST-11 *Neisseria meningitidis.* EBioMedicine 2:1447–1455. 26629539 10.1016/j.ebiom.2015.09.007 PMC4634745

[evw122-B33] Mustapha MM Marsh JW Harrison LH. 2016 Global epidemiology of capsular group W meningococcal disease (1970-2015): multifocal emergence and persistence of hypervirulent sequence type (ST)-11 clonal complex. Vaccine 34:1515–1523. 26876439 10.1016/j.vaccine.2016.02.014

[evw122-B34] Romanow A, 2013 Biochemical and biophysical characterization of the sialyl-/hexosyltransferase synthesizing the meningococcal serogroup W135 heteropolysaccharide capsule. J Biol Chem. 288:11718–11730. 23439648 10.1074/jbc.M113.452276 PMC3636861

[evw122-B35] Romanow A, 2014 Dissection of hexosyl- and sialyltransferase domains in the bifunctional capsule polymerases from *Neisseria meningitidis* W and Y defines a new sialyltransferase family. J Biol Chem. 289:33945–33957. 25342753 10.1074/jbc.M114.597773 PMC4256332

[evw122-B36] Spinosa MR, 2007 The *Neisseria meningitidis* capsule is important for intracellular survival in human cells. Infect Immun. 75:3594–3603. 17470547 10.1128/IAI.01945-06 PMC1932921

[evw122-B37] Stephens DS. 2009 Biology and pathogenesis of the evolutionarily successful, obligate human bacterium *Neisseria meningitidis.* Vaccine 27(Suppl 2):B71–B77. 19477055 10.1016/j.vaccine.2009.04.070 PMC2712446

[evw122-B38] Swartley JS, 1997 Capsule switching of *Neisseria meningitidis.* Proc Natl Acad Sci U S A. 94:271–276. 8990198 10.1073/pnas.94.1.271 PMC19312

[evw122-B39] Taha MK, 2000 Serogroup W135 meningococcal disease in Hajj pilgrims. Lancet 356:2159. 11191548 10.1016/S0140-6736(00)03502-9

[evw122-B40] Tamura K, 2011 MEGA5: molecular evolutionary genetics analysis using maximum likelihood, evolutionary distance, and maximum parsimony methods. Mol Biol Evol. 28:2731–2739. 21546353 10.1093/molbev/msr121 PMC3203626

[evw122-B41] Tibayrenc M Ayala FJ. 2015 How clonal are Neisseria species? The epidemic clonality model revisited. Proc Natl Acad Sci U S A. 112:8909–8913. 26195766 10.1073/pnas.1502900112 PMC4517260

[evw122-B42] Tzeng YL, 2005 Translocation and surface expression of lipidated serogroup B capsular polysaccharide in *Neisseria meningitidis*. Infect Immun. 73:1491–1505. 15731047 10.1128/IAI.73.3.1491-1505.2005 PMC1064937

[evw122-B43] Wang Q, 2010 Genetic study of capsular switching between *Neisseria meningitidis* sequence type 7 serogroup A and C strains. Infect Immun. 78:3883–3888. 20624905 10.1128/IAI.00363-10 PMC2937455

[evw122-B44] Zhou H, 2013 Spread of *Neisseria meningitidis* serogroup W clone, China. Emerg Infect Dis. 19:1496–1499. 23965378 10.3201/eid1909.130160 PMC3810921

[evw122-B45] Zhu B, 2015 Sequence type 4821 clonal complex serogroup B *Neisseria meningitidis* in China, 1978-2013. Emerg Infect Dis. 21:925–932. 25989189 10.3201/eid2106.140687 PMC4451889

[evw122-B46] Zhu P, 2001 Fit genotypes and escape variants of subgroup III *Neisseria meningitidis* during three pandemics of epidemic meningitis. Proc Natl Acad Sci U S A. 98:5234–5239. 11287631 10.1073/pnas.061386098 PMC33193

