# Supplementary material for: Genomic Investigation Reveals Highly Conserved, Mosaic, Recombination Events Associated with Capsular Switching among Invasive Neisseria meningitidis Serogroup W Sequence Type (ST)-11 Strains
Source: Genome Biol Evol. 2016 Jun 11;8(6):2065–75. doi: 10.1093/gbe/evw122 (PMC4943193; doi:10.1093/gbe/evw122)
Supplement: Supplementary Data [file supp_8_6_2065__index.html]

Genomic Investigation Reveals Highly Conserved, Mosaic, Recombination Events Associated with Capsular Switching among Invasive Neisseria meningitidis Serogroup W Sequence Type (ST)-11 Strains — Supplementary Data 

# Genomic Investigation Reveals Highly Conserved, Mosaic, Recombination Events Associated with Capsular Switching among Invasive *Neisseria meningitidis* Serogroup W Sequence Type (ST)-11 Strains

## Supplementary Data

files

- Supplementary Data - xlsx file
